# Beyond the campus: the development of social emotional competence in freshmen through a community-based eldercare volunteer service project

**DOI:** 10.3389/fpsyg.2025.1605930

**Published:** 2025-07-21

**Authors:** Keyi Lyu, Hanxiao Lin, Danlei Zhu

**Affiliations:** ^1^Jing Hengyi School of Education, Hangzhou Normal University, Hangzhou, China; ^2^Chinese Education Modernization Research Institute of Hangzhou Normal University, Hangzhou, China; ^3^Institute of Vocational and Adult Education, East China Normal University, Shanghai, China

**Keywords:** social emotional learning, social emotional competence, college freshmen, intergenerational interaction, community-based volunteer service

## Abstract

Enhancing social emotional competence for freshmen is crucial for their successful integration into diverse university and social environments. While existing research mainly focused on the impact of specific social emotional learning courses on freshmen, there has been limited exploration of the influence of social practice participation. This study investigates the impact of intergenerational volunteer service on freshmen's SEC growth through a collaborative project between H University and G Community from October to December 2024. Through semi-structured interviews with thirteen first-year student volunteers who participated in this project, the findings indicate that participation significantly enhanced freshmen's SEC in five key areas: (1) broaden students' perspectives on university life, (2) reduce study-related anxiety, (3) strengthen social connections and social awareness, (4) promote altruistic behavior and responsible decision-making, and (5) enhance self-confidence and self-efficacy. The results highlight that SEC development is an interactive process involving cognition, behavior, and emotion. Furthermore, eldercare communities can serve as unique informal learning spaces.

## 1 Introduction

The benefits of social–emotional learning (SEL) have been increasingly recognized over the past 30 years. SEL is a process through which children and adults understand and manage emotions, set and achieve positive goals, feel and show empathy for others, establish and maintain positive relationships, and make responsible decisions (CASEL, 2012). SEL has received a surging interest among educators and policymakers for children's development (Jones and Doolittle, 2017).

In recent years, research on SEL has begun to focus on its application in higher education. A high level of SEL can help university students to overcome difficulties and adapt to new life experiences. For instance, SEL is significant in enhancing academic skills, building supportive relationships, managing emotions, and increasing adjustment (Turki et al., 2018). While the importance of SEL for university students has been highlighted, many reports have shown that students are not equipped with the sufficient social–emotional competence (SEC) to deal with the challenges of college life. Unsuccessful mental adjustment is one of the manifestations of this issue. According to the WHO-supported World Mental Health International College Student project, 35% of first-year university students suffer from at least one of the more common lifetime mental disorders (Auerbach et al., 2018). As for China, research has shown that the prevalence of depression, anxiety, and sleep problems among high school students in recent decades has reached 28.0%, 26.3%, and 23.0%, respectively (Yu C. F. et al., 2022).

Recently, researchers have started to focus on exploring how to cultivate university students' SEC. SEL programs have been implemented in various forms, with most innovations focusing on adaptive skills training curricula (Wang et al., 2012) and subject-based curricula (Elmi, 2020). In China, some SEL programs have been carried out in universities attached to subject education, such as mathematics courses, music courses, as well as ideological and political education (Yao et al., 2023; Zheng, 2023). However, SEC is developed not only through formal learning, but also through “doing” (Dewey, 2015). Beyond formal SEL programs, students also develop SEC through undergraduate research, community service learning, learning communities, and other high-impact educational practices (Fink, 2016).

While the importance of social practice in fostering SEC has been acknowledged, there has been little research conducted examining the specific impacts of social practice on SEC and its underlying mechanisms. For instance, Liu and colleagues (Liu and Wang, 2023) found that social practice is more effective for developing the SEC of top students with relatively low ability levels and that it has a significant positive impact on those from low socioeconomic backgrounds. However, those studies often relied on quantitative methods, without exploring the dynamic development of SEC. Therefore, this study aims to investigate whether and how students can develop their SEC through social practice. Through addressing this gap, this study not only extends the discussion on SEL beyond the university classroom, but also offers higher education institutions new pathways for fostering SEC in students.

## 2 Literature review

### 2.1 Innovations for students' SEL in/beyond campus

According to the Collaborative for Academic, Social, and Emotional Learning (CASEL) framework (2020), SEL refers to an individual's ability to understand and manage emotions, establish and maintain positive relationships, and make responsible decisions. These competencies are categorized into five inter-related domains: self-awareness, self-management, social awareness, relationship skills, and responsible decision making (CASEL, 2012). SECs are essential for students' overall development, influencing their emotional wellbeing, academic achievement, and social adaptability.

Beyond the CASEL framework, other conceptual models also emphasize the multi-dimensional nature of SEC development. The “three dimensions and six phases” model from China, along with the Social and Emotional Aspects of Learning (SEAL) framework, categorizes SEC indicators into cognitive, behavioral, and emotional dimensions, underscoring that SECs are not isolated skills but, rather, interdependent constructs that dynamically shape students' personal and social growth (CASEL, 2020). Scholars have highlighted that SECs are particularly critical during higher education, as college students navigate increasing academic pressures, social transitions, and career uncertainties (Mahoney et al., 2018). Developing SECs can enhance students‘ resilience, motivation, and capacity to engage in ethical decision making (Brackett et al., 2019).

While the importance of SEL for college students' development is well-documented, there is a notable scarcity of programs specifically designed for this demographic. Most existing programs are primarily implemented within campus settings, where they are often embedded into courses, mentoring programs, or collaborative activities. These measures focus on enhancing self-awareness, self-management, and emotional adjustment to help students cope with academic pressures and social challenges. For example, Lisciandro et al. (2016) implemented two programs to promote resilience, sustained motivation, and academic self-efficacy among disadvantaged students. Similarly, Chang et al. (2024) designed effective SEL strategies for online classrooms, enhancing academic success and overall wellbeing. Wang et al. (2012) found that SEL seminars improved students' emotional regulation and academic confidence. The “SuccEssfuL in Stats” program reduced anxiety and improved emotional management, enhancing academic performance (Stocker and Gallagher, 2019).

Other programs enhance social skills and emotional intelligence through cognitive remediation therapy, role-playing, group projects, mindfulness practices, team-building activities, and conflict resolution workshops (Arnold-Cotchery, 2023; Clark, 2024; Luckman, 2023). Social-based programs, such as those involving physical activity and social practice, have also shown positive effects on career development, relationships, and emotional health (Simms et al., 2024).

Despite their benefits, most campus-based SEL programs have some limitations: first, these initiatives primarily aim to improve emotional regulation and academic confidence, often neglecting broader aspects such as social awareness and responsible decision making. Second, interactions typically occur within same-age peer groups and classroom settings, restricting students' exposure to diverse perspectives and social experiences. Third, classroom-based interventions fail to provide authentic, complex social scenarios, making it difficult for students to apply their SEC skills in practical situations (Durlak et al., 2011).

### 2.2 Community service as a kind of volunteer service

Compared to traditional campus-based initiatives, volunteer service activities outside campus provide a more diverse and dynamic context for social-emotional learning. According to the United Nations Volunteers, volunteer service involves activities undertaken freely and voluntarily for the common good, without expectation of material reward (Beigbeder, 2023). Volunteering can be classified distinctly based on the primary setting or context of activities into three non-overlapping types: community-based volunteering, which refers specifically to organized volunteer activities aimed at addressing local community needs; international volunteering, involving cross-cultural or global service initiatives; and virtual volunteering, which encompasses online activities that allow volunteers to contribute remotely. In this context, the term “community service” is used interchangeably with “community-based volunteering,” as both specifically refer to organized activities aimed at addressing local community needs. This study focuses on community service, the main type of volunteering among Chinese university students.

Some studies have specifically examined the impact of intergenerational volunteer interactions on SEC development. For instance, civic development theory conceptualizes volunteering as a “civic apprenticeship,” enabling young people to develop empathy, perspective-taking, and collective efficacy through active engagement with community issues (Pavlova et al., 2022). Similarly, cross-national studies guided by self-determination theory indicate that meeting autonomy, relatedness, and competence needs in volunteer contexts promotes sustained emotional regulation skills (Kramer et al., 2021). Empirical evidence supports these theoretical assertions. Large-scale surveys and meta-analyses conducted in over thirty countries demonstrate that volunteering provides students with abundant opportunities to interact with people from diverse ages, cultures, and social contexts, significantly enhancing their SEC (Forner et al., 2024; Nichol et al., 2024; Russell et al., 2023). Coelho and Menezes (2021) reported moderate improvements in empathy, teamwork, and emotion regulation among university volunteers, particularly when programs incorporated structured reflection components and meaningful responsibilities. Additionally, Bringle et al. (2023) found that international service-learning experiences significantly strengthened students' intercultural competence and global empathy.

A limited yet growing body of research specifically addresses the impact of intergenerational volunteer interactions on SEC development. For example, Fong et al. (2024) observed that eldercare services provided college students with meaningful experiences in emotional regulation and empathy through sustained relationships with older adults. Nevertheless, the full potential and detailed developmental processes underlying SEC growth within intergenerational volunteer contexts remain underexplored. Addressing this gap, the present study investigates the processes of SEC development among college students participating in intergenerational volunteer service programs in Chinese universities. Specifically, it examines how students navigate emotional awareness, expression, and regulation within authentic intergenerational community service contexts.

### 2.3 Developing a tentative model of student SEC development in community service

To understand the development of SEC in intergenerational or community-based environments, this study adopts the triadic reciprocal perspective, which is rooted in Bandura's reciprocal determinism theory (Bandura, 1978b). This theory posits that behavior, cognition, and environment interact continuously, shaping one another in a dynamic process. Unlike unidirectional models that view learning as a linear outcome of either individual traits or environmental factors, the triadic reciprocal framework acknowledges that SEC development is an ongoing, reciprocal exchange between an individual and their social context (Pellitteri and Smith, 2007; Bandura, 1978a).

**Figure 1:**
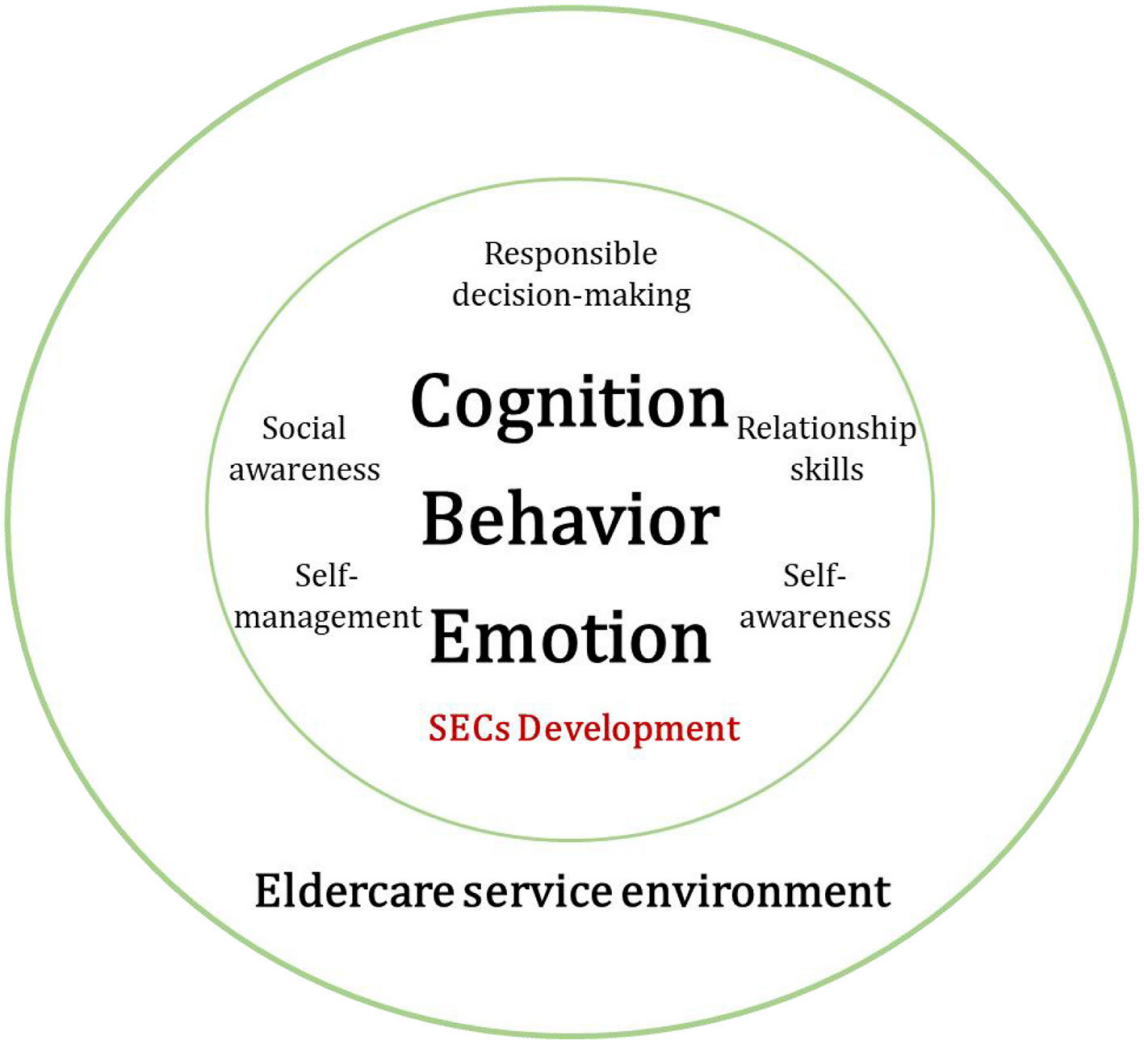
A model of SEC development in students through community eldercare services.

The adoption of the triadic reciprocal perspective in this study is based on the following justifications:

1. SEC is context-dependent and socially constructed: traditional SEL interventions often assume a linear progression of skills, where competencies are built incrementally through structured training. However, SECs are not just acquired passively; they are actively negotiated and shaped through social interactions (Zins et al., 2007). Bandura's model helps illustrate how students internalize, modify, and apply SECs in response to their real-world experiences.
2. SEC development is influenced by multiple interacting factors: the triadic framework highlights how cognitive, behavioral, and environmental factors continuously reinforce each other. For instance, a student's self-perception of their ability to communicate with older adults (cognition) influences their engagement in conversations (behavior) which, in turn, is shaped by the responses and feedback they receive from the community (environment) (Bandura, 1977).
3. Real-world engagement enhances SEC application: unlike controlled classroom-based training, community service exposes students to unstructured, unpredictable social settings. The triadic reciprocal model accounts for the adaptive nature of SECs, where students must continuously adjust their behavior and emotional responses based on their evolving experiences within the community.
4. Building upon this theoretical foundation, we propose a provisional framework for understanding how students develop SEC through community service, particularly within eldercare settings.
5. Figure 1 outlines how cognitive, behavioral, and emotional dimensions interact dynamically in SEL within eldercare service environments. The model employs the CASEL framework to assess changes in SEC development over time. The diagram il-lustrates how community eldercare engagement reinforces SEC growth by offering authentic social experiences, promoting ethical decision making, and fostering deeper social awareness and emotional intelligence.

Integrating the international volunteer-service lens with the CASEL framework leads us to treat eldercare volunteering not merely as “off-campus practice,” but as a developmental arena where SECs, civic identity, and intergenerational solidarity co-evolve. By positioning SEC development within a reciprocal, contextually embedded learning process, this study aims to provide a more comprehensive understanding of how students cultivate resilience, empathy, and social responsibility through inter-generational service experiences. By positioning SEC development within a reciprocal, contextually embedded learning process, this study aims to provide a more comprehensive understanding of how students cultivate resilience, empathy, and social responsibility through inter-generational service experiences.

## 3 Materials and methods

### 3.1 Study context

Freshmen have shifted from the “exam-centric” high school environment and the protective setting of their original family to the diverse and challenging environment of university life. Some students may soon “free themselves,” enjoying communal activities and leisure; others may feel lost, bewildered, and lonely in the new surroundings because they left their hometowns without emotional support. A 2024 survey claims that over 70% of first-year university students go through emotional swings and have problems in their relationships (Song and Hu, 2024). As students struggle for scarce high-quality educational resources, some of the “Gaokao system” champions enter colleges and develop a strong competitive academic mindset (Zhou et al., 2022). A notable example of this phenomenon is the emergence of the term “小镇做题家” (literally, “small-town exam warriors”) in China in recent years. This term describes pupils who, at the expense of ignoring chances for personal development, social contacts, and emotional adaptation, reach academic success through extremely focused, exam-centric approaches (Li, 2024).

A survey (Gao, 2022) on the status quo of social–emotional abilities among Chinese college students indicates that university students have already shown some cognitive level development. They have built self-awareness, good personal cognition, and a wish to see the larger world. However, given the restriction of their mental developmental stage, their attitude remains immature, and their resilience needs to be improved. Students are immersed in an intense, exam-oriented learning environment with the ultimate goal of “Gaokao” before they start university. When they become university students, they often refer to this period as “the peak of my intelligence.” Indeed, under the demanding requirements and peer pressure of high school, students show substantial improvement in academic self-management. Still, some Chinese students often believe that work, not aptitude, is the main path to success (Stankov, 2010), which causes them difficulty in escaping this one definition of success and thus only consider high marks as valuable. This academic worry affects not only their mental state, but also prevents the complete growth of their SEC.

Since their time is mostly devoted to classroom learning and extracurricular tutoring, this academic-oriented high school life sometimes limits students' chances to interact with more general society. Chinese students devote an average of 57 h per week to academic activities—significantly more than the global average of 44 h (OECD, 2019). They might thus lack a vivid grasp of social dynamics, which would make it difficult for them to adapt to complex social environments.

Additionally, many students come from middle-class and lower-income families, where financial constraints and time limitations may restrict their access to diverse cultural experiences. According to the China Education Panel Survey (Institute of Social Science Survey, 2015), only 35% of students from lower-income families participated in extracurricular activities compared to 60% of students from higher-income families. This limited exposure to broader societal contexts further compounds their challenges in developing a comprehensive understanding of global issues and societal norms.

The exam-oriented and classroom-centric environment prevalent in high schools imposes significant limitations on the development of students' social awareness, interpersonal relationships, and sound decision making. As students transition from the sheltered environment of the “ivory tower” to real society, they need to break away from a narrow “student mindset” and adopt an “adult mindset” that is characterized by active exploration, critical reflection, and independent decision making. The deep integration of society and mature development of one's mindset, as well as positive responsibility, are all advanced manifestations of SEC. Therefore, social–emotional learning for college students should extend beyond the traditional classroom, incorporating programs that transcend conventional education and foster competencies for future development. Particularly, volunteer service projects—targeting diverse groups such as the elderly, children, and people with disabilities—create dynamic social settings that facilitate interactions across different social groups, help participants manage unexpected events, and prompt them to re-evaluate their own roles and society at large. These encounters notably help students to develop social–emotional ability. According to certain research, community service greatly improves university students‘ sense of social responsibility, self-awareness, and knowledge of society concerns (Astin et al., 2000; Youniss, 1996). Thus, community service could be a particularly important pragmatic tool in higher education to advance the growth of SEC.

### 3.2 Project design

This qualitative study was conducted in Hangzhou, the capital city of Zhejiang Province, China. With 24% of its population aged 60 and above, Hangzhou is experiencing moderate population aging (Hangzhou Municipal Bureau of Statistics, 2023). To promote active aging, efforts are being made to establish age-friendly communities and foster partnerships between universities and community organizations. This context provides an ideal environment for exploring intergenerational programs, particularly as Hangzhou's educational resources, with nearly 50 universities and a high college student population, offer ample opportunity for volunteer involvement in community service (Hangzhou Municipal Bureau of Statistics, 2022).

For this project, it took place from October to December 2024 and involved a partnership between H University and G Community. This volunteer service team was founded in 2003 and has grown steadily over the past 22 years. Its intergenerational activities focus on themes such as art, the use of smart devices, healthcare and wellness, and anti-fraud education to support older adults. Every year, around 200 volunteers participate in the project, bringing in new energy and perspectives.

Volunteers, primarily freshman students from H University, were recruited and trained under the guidance of experienced volunteer leaders. Volunteer leaders informed recruiting message in the Ding Talk group of H university students 1 month in advance, so that students could know the service time, place, content and the volunteer hours they could accumulate. And then the members from volunteer association screened out people on the blacklist (people who affected any volunteer activities for extremely bad reasons). Three weeks before the event starts, students on short list were trained. The training content are (1) learning service concept and rules: they were introduced service activity's basic information, service request, rules and the consequences of violating the rules. (2) Knowing how to serve elder people: through the sharing of experienced team member and group talking, volunteers could know more about the older people, anticipate the possible situation and countermeasures. (3) Familiarizing service process and personal work: Service process was informed to students, and tasks were allocated to them based on their abilities and wills. Training lasted 1-week, equipping students with fundamental skills in eldercare and preparing them for the unique dynamics of intergenerational interaction.

Throughout the program, students participated in three monthly birthday celebrations for older adults, aged between 70 and 80, in G Community. These 60- to 75-min events combined entertainment and social engagement, including singing, dancing, crosstalk performances, and interactive games to foster lively interaction. Occasionally, educational lectures were offered for the older adults, providing both cognitive stimulation and enjoyment. Each celebration concluded with the volunteers singing “Happy Birthday,” presenting cakes and long noodles as symbols of longevity, creating a warm and respectful atmosphere. The activities were led by volunteer leaders and community practitioners, while the freshman volunteers took active roles, following protocols designed to ensure positive, respectful engagement.

This study included a diverse group of participants: 13 freshman volunteers (aged 18 to 20, including 12 females and 1 male), 30 older adults from G Community, 3 volunteer leaders, and 1 care home activity practitioner. Given that the freshman volunteers had minimal experience with elderly care, the training and program activities were designed to ease them into their roles while promoting intergenerational bonding and SEC development. This study, initiated by H University, was structured to promote meaningful intergenerational interactions and observe their impact on the social–emotional growth of college students.

### 3.3 Data collection and analysis

Approval for this study was obtained from project managers, and written in-formed consent was collected from all participants, with pseudonyms being used to protect individual identities. We represent each research subject in the form of “gender – number.” For example, “M-1” stands for the male with number 1, and “F-2” stands for the female with number 2 (see Table 1).

**Table 1:** The participants.

Code^*^	**Major**	**Main tasks**	**Age**
F-1	Primary education	Volunteer management, host, game planner	19
F-2	Primary education	Volunteer management, host, prop fabrication	19
F-3	Primary education	Sang songs, gave out birthday cakes	18
F-4	Primary education	Volunteer management, host	19
F-5	Preschool education	Volunteer management, host	19
F-6	Primary education	Sang songs, gave out birthday cakes	19
F-7	Practical psychology	Host, sang and danced	20
F-8	Nursing	Performed skits, sang songs	19
F-9	Primary education	Sang songs, recited	19
M-10	Educational technology	Prop fabrication, game planner, volunteer management	18
F-11	Preschool education	Leading volunteer, danced, performed skits,	19
F-12	Preschool education	Sang and danced	19
F-13	Primary education	Leading volunteer, danced, sang songs	19

^*^F, female; M, male.

The researchers followed the changes in the behavior and attitudes of the college student volunteers during three volunteer sessions, and they also participated along-side them. In the first month, the second author primarily recorded the volunteers' attitudes, behaviors, and specific events during the service. Subsequently, the research team explored questions that emerged from these early observations. As the research hypotheses became clearer, the observations focused more specifically on college students' self-awareness, self-management, social awareness, prosocial behavior, and responsible decision-making during their interactions with older adults. Considering the privacy concerns of both the older adults and the young volunteers, we did not audio-record the entire process.

Preliminary discussions were held after each activity. In-depth interviews were conducted with 13 volunteers in December 2024. Each one-on-one onsite conversation lasted for half an hour and was recorded. The language used in the interview process was Chinese. After examining interview materials, the materials and analysis result were translated into English and then use the back-translation method to improve the accuracy of the language.

This study drew on the CASEL framework for Social and Emotional Learning (CASEL, 2020), to design the interview protocol, capturing the impact of volunteer service on the volunteers' SEC across five dimensions: self-awareness, self-management, social awareness, relationship skills, and responsible decision making. To uncover the dynamic process of SEC development, the interviewees were invited to recount memorable key events and reflect on their cognitive and emotional changes. This research employed participant observation alongside semi-structured interviews. As Patton (2015) and Charmaz (2006) pointed out, combining multiple data sources enables researchers to conduct cross-validation and capture subtle details that are easily overlooked in interviews alone. The observational data revealed actual behaviors and emotional responses, effectively supplementing the verbal data obtained through interviews. The final research findings organized by theme are shown in Table 2.

**Table 2:** Illustrative quotes, initial themes, and final thematic categories.

**Original quote**	**Initial theme**	**Final thematic category**
(Participating in this activity) made me realize that university life should not only stay in the college. It is important to meet and contact people. This not only releases pressure but also enhances communication skills. (M-10)	Stepping outside the campus	Promoting a more open perspective on university life
I thought there was a big gap between their lives and ours. However, after contacting with them, I realized that the differences are more about physical convenience. For example, during our performances, they would even record us with their phones. It was quite interesting. (M-11)	Reduction of stereotypes	
Grandparents have gone through so much yet remain so cheerful, which makes us, the younger generation, more optimistic about the infinite possibilities of the future. (F-8)	Optimistic outlook on the future	Reduction in study-related anxiety
The patience and kindness of the older adults make me feel warm and relaxed. Every interaction helps me understand myself better and learn how to cope with stress. (F-12)	Emotional support	
After finishing the volunteer work and walking through the community, I felt like I had returned home. (F-9)	Sense of home and belonging	Fostering positive connections and social awareness
At that time, I felt that interacting with them led me to discover that some of the elder people were quite interesting. They seemed genuinely interested in smartphones and were very willing to learn. (F-6)	Discovering the interests of older adults	
“I noticed that some grandparents appeared to be somewhat lonely and in need of more companionship and care. These (volunteer service) experiences have made me better understand and care for those around me, especially the elder.” (F-3)	Recognizing the situation and real needs of the older adult	
The volunteers also began to think about how their studies and work can serve older people. A nursing volunteer also expressed a desire to cultivate his professional skills so that he could better serve older people in the future and pay attention to this group's medical needs (F-5).	Exploring potential for professional elderly services	
For example, in my dormitory, I often encourage my roommates to participate in activities together, inviting them to join us in serving the older people. (F-1)	Calling on peers to participate	Encouragement of altruistic behavior and responsible decision making
This activity influenced my thoughts about my grandparents. Since high school, I have rarely spent time with them. After completing my volunteer work, I decided to call them whenever I had the chance (F-7).	Emotional connection with grandparents	
I think some elder people come here primarily to receive flowers or other items rather than to genuinely enjoy the performances. I hope that through our future volunteer activities, they will find these programs truly engaging and participate actively, not just for the sake of receiving gifts (M-11).	Reflecting on and improving the activity	
“I had no opportunity to stand in front of so many people before, but now I can host on stage. Although it is a simple task, I consider it a form of practice for me. I have social anxiety, but seeing the kindness of the older people made me more willing to express myself.” (F-1)	Willingness to express oneself publicly	Enhanced confidence and self-efficacy
“They [volunteers] are all enthusiastic and dedicated, and have some talent. This is a great opportunity for communication and interaction.” (F-8)	Empathizing with the role of fellow volunteers	

Qualitative thematic analysis was then adopted to identify various changes (Patton, 2015; Hammarberg et al., 2016). The following sequential steps were followed: (1) All authors read and familiarized themselves with the interview data; (2) The second author generated initial codes independently; (3) The first author and second author check and compare the codes; (4) Themes were derived and cross-checked by all authors; (4) All authors reviewed, negotiated, and defined the emerging themes (Saldaña, 2021; Braun and Clarke, 2023). According to Fereday and Muir-Cochrane (2006), an iterative and reflexive process is necessary for promoting and demonstrating the rigor of thematic analysis. From Steps 3 to 5, the authors conducted continuous comparisons, as well as discussions and reflection on the codes. In this process, the authors also invited a supervisor to code the data. The results were compared, and no further modifications were required.

## 4 Findings

### 4.1 Promoting a more open perspective on university life

Eldercare service projects significantly influence students' perceptions of university life, encouraging them to adopt a more open approach to social and academic activities. Some freshman volunteers noted that without participating in such activities, their university life schedule might have only been filled with academic tasks, campus activities, and limited social interactions with roommates, as they would have lacked the motivation to explore broader horizons. One student (M-10) shared the following reflection:

“(Participating in this activity) made me realize that university life should not only stay in the college. It is important to meet and contact people. This not only releases pressure but also enhances communication skills.” (M-10)

Volunteers recounted numerous heartwarming and surprising moments during their service. For instance, “grandmothers” singing along to music, “introverted grandfathers” subtly clapping to the rhythm, and the ambivalence of the “older grandfather” trying new activities while fearing embarrassment. One particularly funny moment was when a “grandfather,” out of curiosity, gently patted another's bald head, sparking laughter among everyone present. This anecdote was mentioned by more than one volunteer. At the beginning, they were surprised about the contrast between behavior and age. In order to take care of the overall situation, they hid their belly laughter in tiny dimples on their face and continued to finish the service task, while also being unable to not think about the ridiculous experience. It is true that their preconceived notions about older adults were challenged by real interactions that dispelled stereotypes. These genuine interactions not only changed the volunteers' perceptions of older people, but also ignited their enthusiasm for exploring life beyond campus. These experiences enriched their emotion and motivated them to actively engage in social activities, breaking down the boundaries between campus and society.

Repeated interactions with the community encouraged students to openly express their emotions during performances and to stimulate their passion more for university life. Some volunteers mentioned that they began to participate more actively in campus activities, no longer satisfied with a fixed routine of studying and socializing. Additionally, brief coexistence with older adults reshaped volunteers' perceptions of aging. Research has shown that natural intergenerational interactions can reduce stereotypes about aging among college students (Teater, 2018), and this study reaffirms this finding. During these interactions, the genuine and childlike nature of older adults often touched the volunteers deeply. One student (M-11) reflected on this as follows:

“I thought there was a big gap between their lives and ours. However, after contacting with them, I realized that the differences are more about physical convenience. For example, during our performances, they would even record us with their phones. It was quite interesting.” (M-11)

In the context of eldercare services, time perception is disparate compared to the rigid structure of campus life, triggering life course reflection (Elder, 2019) among students. One volunteer (F-6) described “playing mahjong and chatting is the ideal older people life,” imagining a future where time is experienced more meaningfully. On the other hand, other volunteers became aware of the finiteness of time, which also shifted their life goals toward “meaningful experiences” (Carstensen, 2006). Time was no longer simply divided into “study & rest” or “success & failure.” On the other hand, the resilience and optimism of the older people deconstructed the negative social label of aging, allowing young people to seize the possibilities of life. Volunteers often expressed sentiments such as the following:

“Grandparents have gone through so much yet remain so cheerful, which makes us, the younger generation, more optimistic about the infinite possibilities of the future.” (F-8)

Seeing the lifestyles of older adults, young people projected their future selves onto a more positive mirror. They began to see university life as an exploratory journey, motivating them to cherish their time and try new things, rather than viewing it as a linear race.

### 4.2 Reduction in study-related anxiety

As the competition in higher education is intense, freshmen commonly face academic anxiety and psychological adaptation crises (Song and Hu, 2024). The Academic Incompetence Hypothesis suggests that academic failure can lead to low self-efficacy and emotional distress, as well as externalized problems, like social difficulties (Song et al., 2024). Eldercare volunteer programs provide an emotional support platform for freshmen, alleviating their academic anxiety through emotional bonding and fostering a sense of belonging.

Eldercare services offer volunteers an opportunity to step away from the competitive academic environment. Unlike the performance-oriented atmosphere of university classrooms, eldercare programs emphasize creating a lively and inclusive environment rather than technical perfection, hence creating a pressure-free space for interaction. One volunteer (F-12) described this as follows:

“The patience and kindness of the older adults make me feel warm and relaxed. Every interaction helps me understand myself better and learn how to cope with stress.”

According to the Social Support Buffering Theory (Cohen and Wills, 1985), in stressful situations, social support compensates for psychological resource deficits by providing emotional, informational, and evaluative resources. Through intergenerational interactions, volunteers gain emotional support. This support focuses on the volunteers' “kindness” rather than their “competence,” which could be characterized by non-utilitarian qualities. Such support compensates for the lack of emotional resources in the university, helping students temporarily detach from academic anxiety and rebuild emotional balance.

For freshmen studying away from home, community eldercare activities provide a sense of “home” through a relaxed atmosphere and familial affection. One volunteer shared the following reflection:

“After finishing the volunteer work and walking through the community, I felt like I had returned home.” (F-9)

This grandparent-like care not only reduce loneliness, but also enhances emotional stability and a sense of belonging. Maslow (1943) regarded the need for belonging and love as a fundamental human need. When individuals feel a sense of belonging, they experience emotional security and support, which enhances psychological resilience (Song et al., 2024). This enables them to adopt a more positive attitude toward setbacks, reducing emotional fluctuations and, ultimately, helping one to face academic challenges with greater confidence.

The pace of eldercare activities contrasts sharply with the fast-paced campus life. Due to the hearing impairments of some older individuals, volunteers would bend down, speak slowly, and articulate clearly. When organizing games, volunteers patiently waited for the “grandparents” to complete their actions. Gradually, volunteers adapted to this slower pace, temporarily escaping the pressures of modern life. Through activities such as games, riddles, and storytelling, the volunteers not only received positive feedback, but also experienced genuine emotional connections and warmth. This slower pace provided a psychological buffer, allowing students to face academic pressures with a calm mindset.

### 4.3 Fostering positive connections and social awareness

In the warm community-based eldercare services, social relationships are not merely passively established; instead, they are actively formed and deepened through listening, communication, attention, and cooperation. These connections not only exhibit a superficial harmony, but also reflect the update of individual identity and the awakening of social awareness.

Volunteerism simultaneously embodies both organization and spontaneity. The content, format, sequence, and participants of the programs were well managed; the themes of birthday parties and game sessions were pre-determined. However, for the freshmen participating in eldercare services for the first time, they were still adapting to their roles as volunteers and felt a sense of unfamiliarity and distance toward the lives of the older adults. These students often enter the service environment with a tentative attitude, trying to discern the specific ‘missions' of volunteers and the actual needs of the older adults. As interaction increases, the initial gap is gradually replaced by a subtle and genuine mutual understanding. This understanding does not occur overnight but slowly ferments through seemingly trivial conversations and practices.

The theme activity ‘Decrease the Intergenerational Digital Divide' fostered closer relationships between the volunteers and the older people. According to the head of the older people care service, these older people individuals belonged to the lower middle class, with lower educational levels, and had engaged in manual labor, such as working as manual workers and farmers, when they were younger. Most of their children did not live with them, and they faced difficulties using phones and other electronic devices.

During the service, a volunteer leader stood on stage as lecturer, while other volunteers assisted the older people through one-on-one teaching. The older people were required to take pictures of their favorite programs and participate in a popular vote to select the most favored works. Volunteers broke down the photography skills into simple steps, teaching the older people how to open the camera function, focus on their subjects, and press the shutter button to take beautiful photos. Initially, the volunteers were worried that the activity might be too simple and lead to a lack of engagement, but the older people actively cooperated with the volunteers to complete the learning tasks, despite facing some challenges.

The volunteers were also surprised by the low prevalence of smartphone use among the older adults. What moved the volunteers even more was that the elder people would spontaneously take out their phones to capture the beautiful moments of the event and proudly share their achievements with the volunteers at the next couple of events.

“At that time, I felt that interacting with them led me to discover that some of the elder people were quite interesting. They seemed genuinely interested in smartphones and were very willing to learn.”(F-6)

This process not only enhances young people's sensitivity toward the older people in society, but it also prompts them to re-examine themselves. The volunteers gradually realized that they were not merely service providers, but also integral parts of the social operations. By directly observing the real living conditions of the elder people, they were able to extend these observations to a broader social context by considering the overlooked situations and needs of the older adults. One volunteer told us the following on this notion:

“I noticed that some grandparents appeared to be somewhat lonely and in need of more companionship and care. These (volunteer service) experiences have made me better understand and care for those around me, especially the elder.” (F-3)

The volunteers also began to think about how their studies and work can serve older people. A nursing volunteer also expressed a desire to cultivate his professional skills so that he could better serve older people in the future and pay attention to this group's medical needs (F-5).

### 4.4 Encouragement of altruistic behavior and responsible decision making

Classical sociological studies have pointed out that the generation of altruistic behavior is not merely a product of moral concepts, but also a result of the individual's perception of social roles (Durkheim and Halls, 2008). In the initial stages of volunteer service, university students often participate with a utilitarian mindset, aiming to “accumulate experience” or “enhance their resumes.” At this stage, their perception of the elder people is limited to stereotypes of “frailty,” and their actions are understood as just involving material care and superficial interactions, such as giving gifts and making older people laugh.

However, as volunteers establish genuine emotional bonds with older people through long-term companionship, their motivation shifts from “passive execution” to “meaning creation.” An increasing number of the volunteers came to realize that the core need of the older people is sincere companionship (F-13). One volunteer reflected on this during an interview as follows:

“These activities made me realize that what grandparents need is not just gifts from us but someone who truly listens to them.”(F-4)

The shift in role perception prompts volunteers to transition from being “service providers” to “companions” (Mead, 2002). When volunteers act as companions, the older people can better sense their sincerity and kindness. This transformation turns one-way giving into a two-way emotional exchange, fostering the development and dissemination of altruistic behavior.

On campus, the volunteers all agreed that eldercare services need more youthful energy. As a result, many of the volunteers invited their roommates and friends to join, hoping to share the relaxed and pleasant atmosphere of the community. One volunteer mentioned: “For example, in my dormitory, I often encourage my roommates to participate in activities together, inviting them to join us in serving the older people” (F-1). Additionally, many volunteers choose to share their experiences on WeChat Moments. In this way, individual altruistic choices gradually evolved into collective actions through the dissemination of social networks and social reality. Students also constructed a new narration within the campus community, such as “volunteer service is not a sacrifice but a two-way healing.”

Such service experiences also trigger reflections on family relationships and emotional responsibilities. When caring for the older people individuals who were similar in age to their own grandparents, the volunteers often projected the emotions generated through intergenerational interactions onto their family members. One volunteer stated the following:

“This activity influenced my thoughts about my grandparents. Since high school, I have rarely spent time with them. After completing my volunteer work, I decided to call them whenever I had the chance” (F-7).

The sense of responsibility cultivated in the nursing home extends to daily life, with volunteers noting that they now remind their grandparents to take medication and visit doctors (F-8).

Furthermore, volunteers have begun to see themselves as active roles in fostering social responsibility, critically examining the current mode of eldercare services. For instance, one volunteer pointed out:

“I think some elder people come here primarily to receive flowers or other items rather than to genuinely enjoy the performances. I hope that through our future volunteer activities, they will find these programs truly engaging and participate actively, not just for the sake of receiving gifts” (M-11).

This critical perspective highlights the need to design activities that link more closely with the inner desires of elder people.

### 4.5 Enhanced confidence and self-efficacy

Compared to university evaluation systems, such as grade point averages and comprehensive assessments, eldercare communities provide students with “non-utilitarian feedback,” fostering an improvement in their self-efficacy. Eldercare service encourages students to take on new roles and develop diverse skills, learning dancing, organizing games, hosting warm-up activities, and developing other multiple skills, thus helping them showcase latent abilities.

These activities are primarily performative, requiring direct interpersonal engagement and acquiring immediate, positive feedback from older adults. This supportive environment encourages volunteers to step beyond their comfort zones. After the program, eleven students (85%) reported feeling “surprised at what I could do,” indicating a broad fulfillment of new capabilities. Specifically, nine volunteers (69%) said that hosting, acting, or dance sessions helped them unlock previously unknown communication or creative skills. Five (38%) noted they could now “generate new ideas quickly” when planning activities, while four (31%)—who had labeled themselves socially anxious at the outset—successfully led a team or coordinated an intergenerational game during at least one service session. As a volunteer expressed:

“I had no opportunity to stand in front of so many people before, but now I can host on stage. Although it is a simple task, I consider it a form of practice for me. I have social anxiety, but seeing the kindness of the older people made me more willing to express myself.” (F-1)

Unlike peers, feedback from older adults is non-utilitarian, allowing young volunteers to enhance their self-efficacy without the pressure of social comparison (Festinger, 1954). Most volunteers used words like “positive” and “enthusiastic” to describe the older adults' feedback. Via the observation of explicit behaviors, like eye impressions, smiling, clapping, etc., we were able to find that the older adults preferred lively skits and game shows. However, they were also obsessed with the beautiful dance program that was conducted and actively clapped along with old Red Army songs or golden classics, though they did not continue to do so when a pop song they were not familiar with and could not understand was played. As long as the performers had a good attitude, they always gave positive feedback. The evaluation “good” was not based on objective or harsh criteria but only subjective feeling. After one event, two old adults kept saying they were “very happy” and gave us some of their snacks. Their goodwill served as a “psychological shock absorber,” reducing the stress accumulated from competitive academic environments and transforming their unrecognized potential into confidence in attempting the unknown.

Group volunteering activities are more likely to facilitate vicarious experiences. The volunteers observed contrasts among their peers, such as a seemingly unremarkable classmate delivering a stellar skit performance on the community stage, earning applause from the older people. The volunteers also appreciated each other's efforts:

“They [volunteers] are all enthusiastic and dedicated, and have some talent. This is a great opportunity for communication and interaction.” (F-8)

In addition to the contrast of peer students in performance, the volunteers were more capable of noticing the meticulousness, overall leadership ability, and calm problem-solving responsibility of their peers during the service participation process. Simultaneously, they were able to more clearly realize their empathy, responsibility, and problem-solving abilities as more precious virtues. This human-centered perspective would only help them to better appreciate the growth of their peers and to be aware of their own progress. Witnessing the progress of others, along with flowers and applause, inspired the volunteers to step out of their comfort zones. This environment fostered a growth mindset among the volunteers, making the freshmen more likely to seize future opportunities. These positive experiences serve as tangible evidence of their personal breakthroughs.

## 5 Conclusion and discussion

This study validated McKay-Jackson's (2014) proposition that community-based service participation fosters holistic SECs through practical cases. Specifically, our findings indicated that engagement in intergenerational service can broaden students' perspectives on university life, reduce study-related anxiety, strengthen social connections and social awareness, promote altruistic behavior and responsible decision making, and enhance self-confidence and self-efficacy.

Moreover, as Figure 1 shows, SEL is not a linear accumulation of skills but, rather, a dynamic and integrative process where individual transformations in cognition, behavior, and emotions interact and reinforce each other. Specifically, we identified three key transformation pathways through which SECs are cultivated in eldercare volunteering: cognitive Schema Renewal, Emotional Ethics Formation, and Action Pathway Expansion.

Intergenerational contact serves as a catalyst that transforms students' cognitive schemas, renewing their perspectives on life, learning, and success. Moving beyond the university setting, the students began to see social engagement as a meaningful learning experience and developed a long-term future perspective (Seijts, 1998) that fosters resilience and optimism. A key finding was the shift in the perceptions of aging: from initial stereotypes of ‘frail' or ‘rigid' older adults to more energetic and engaged older adults. This transformation fits the intergroup contact hypothesis (Allport et al., 2015), and also reflects the students' shift from detached observers to engaged companions. The experience also challenged the traditional “service provider–recipient” mode as the students recognized the relational needs of the older adults, interpreting volunteering as a mutual exchange (1985).

Intergenerational volunteering fosters a unique emotional learning environment. The non-utilitarian feedback and delayed responses from older adults (Chen et al., 2024; Sobczak and Bunzeck, 2023) create a special mechanism of emotional education. Unlike peer interaction (Zhang, 2008), intergenerational exchanges provide a judgment-free space where students receive unconditional appreciation, like a heartfelt smile and spontaneous applause, such that the students were alleviated of their performance anxiety and saw their fear of failure reduced. As the students subconsciously saw the older adults as their grandparents, the students developed a sense of security and belonging (Yu X. et al., 2022), easing their adaptation to new environments. More importantly, through sustained interactions, they witnessed that their actions can really impact older adults' wellbeing. Furthermore, over the course of the study, this emotional resonance was transformed into a deepened sense of social responsibility (Kessler and Staudinger, 2007; Silverstein et al., 2025).

Eldercare volunteering reshaped the students' action logic by immersing them in real-world interactions that demanded adaptation and initiative. Unlike structured learning, volunteering requires reciprocal communication (Manatschal and Freitag, 2014), where students learn to observe unspoken cues and engage in empathetic dialogue. Organizing activities trained their strain capacity as they navigated unexpected challenges and dealt with problems (Fletcher and Sarkar, 2013). Sustained engagement also prompted the students to think about the social difficulties from individual services, strengthening social personality.

While previous studies have explored SEL among peer groups, this study shed light on the functions of intergenerational interactions in SEC development. Unlike peer interactions, which are often shaped by academic competition and social comparison, cross-age engagement creates a psychologically safe space where students feel free to express vulnerability and achieve personal growth. The genuine mutual recognition between students and older adults encourages students to build confidence outside of the academic enclosure.

This study highlights the potential of community-based volunteer service when compared to formal education (Wang et al., 2012; Wyatt and Bloemker, 2013). Unlike structured classroom-based SEL (Durlak et al., 2011), which often relies on fixed theoretical materials, community-based service-learning exposes students to unpredictable real-world situations. There are dual benefit structures from eldercare service: students simultaneously relieve their academic stress while also acquiring emotional support. The volunteers faced true societal challenges from taking into account age-related physical limitations when reconciling intergenerational value conflicts. This type of unscripted human interaction fostered altruistic behavior and critical reflection that transcends book learning. Such experiential learning is in line with Kolb's (1984) experiential learning cycle, where a person grows up through the combination of concrete practice and reflective observation. Community engagement, therefore, serves as a valuable complement to class-based SEL education, helping bridge the theory–practice divide and improving students' SEC.

## 6 Limitations and future research

The first limitation to consider is that, although the volunteer service improved students' SEC, it was restricted by the short-term intervention design (three activities, up to a total of 3 to 4 h). The Hawthorne effect could not be excluded; that is, that the participants altered their behavior simply because they knew they were being observed (McCarney et al., 2007). According to the Self-Determination Theory (Ryan and Deci, 2000), the maintenance of intrinsic motivation requires a supportive environment that fulfills individuals' basic psychological needs. Considering that this study aims to deeply explore the developmental processes of university students through intergenerational interactions, we deliberately focused on a small cohort of 13 first-year students out of 17 participants. Aligns with the goals of qualitative research (Creswell, 1998), the findings are intended to offer in-depth insights and emphasizes the contextualized meanings of participants' experiences, rather than broad generalizations. As an exploratory effort, future research should employ larger sample sizes and hybrid methodologies to validate and extend these initial findings.

The second limitation concerns the gender imbalance among participants, as the majority of volunteers in this study were female. This may partly reflect the student demographics of H University—a normal university where approximately 73% of the student population is female. Previous research showing females are more likely to engage in volunteer work due to higher empathy and prosocial orientation (Einolf, 2011; Eagly, 2013). However, this gender dominance may introduce bias, as emotional responses and development pathways could differ across genders. Future research should aim to achieve a more balanced gender sample to examine whether male students experience similar or different effects.

Third, this study mainly focused on relatively healthy older people in the community; thus, it did not cover high-risk groups, such as those with advanced age (80+), those living alone, and those with disability, resulting in limited external validity. High-risk groups may have higher care needs, which may trigger different mechanisms for SEC development. Future research should enlarge the range of study through stratified sampling (Bronfenbrenner and Ceci, 1994).

Although the data provided in this study describe the process and outcomes of developing SEC, there remains a certain degree of a “black box” phenomenon in revealing the underlying mechanisms. Specifically, existing research has not yet clearly answered the following key questions: among the various interactive elements, which are the most critical? How does a student's initial SEC level moderate the intervention effects?

The available data did not fully reveal specific mechanisms, such as the key elements of the interactions and how a student's original SEC would affect the transformation. Future studies need to focus on aspects such as student gender, initial SEC status, and the characteristics of the service recipients, and also need to adapt mixed methods to explore this phenomenon more deeply.

## Data Availability

The raw data are retained by the authors to protect participant confidentiality. Data may be made available upon reasonable request via email to the corresponding author, with a formal explanation of intended use.
